# Potential Applications of Nanomaterials to Quench the Cytokine Storm in Coronavirus Disease 19

**DOI:** 10.3389/fbioe.2020.00906

**Published:** 2020-08-19

**Authors:** Anissa Pisani, Pier Paolo Pompa, Giuseppe Bardi

**Affiliations:** ^1^Nanobiointeractions & Nanodiagnostics, Istituto Italiano di Tecnologia, Genoa, Italy; ^2^Department of Chemistry and Industrial Chemistry, University of Genova, Genoa, Italy

**Keywords:** cytokine storm, inflammation, Covid19, nanomaterials, chemokines

## Introduction

Sars-Cov-2 virus infection induces coronavirus disease 19 (Covid19) with severe prognosis in weaker individuals. Acute respiratory distress syndrome (ARDS) and multiple-organ failure are the major causes of death in the later stages of the disease (Ramanathan et al., 2020). These poor outcomes follow a general unbalance of the immune response with uncontrolled inflammation and large infiltration of activated leukocytes in the lung and some other organs, including the Central Nervous System (CNS) (Baig et al., 2020). The immunological profile is characterized by the reduction of CD4^+^ and CD8^+^ T-lymphocytes (lymphopenia), decreased level of CD4^+^cell expression of INF-γ and increased cytokines Interleukin-6 (IL6), IL-10 and tumor necrosis factor-alpha (TNFα) (Pedersen and Ho, 2020). Although IL-10 has anti-inflammatory effects, the enormous release of pro-inflammatory mediators overwhelms the feedback mechanisms that regulate innate immunity causing a “cytokine storm.” Actually, several steps of the immune response to the virus are impaired during Covid19. Lymphopenia underlines a compromised adaptive response, while innate immune phagocytes, such as neutrophils and macrophages, invade and damage the lung and the juxtaposed endothelial tissue. This vicious feedforward loop is enhanced by phagocyte chemo-attractant cytokines (chemokines), like CCL2, CCL3, and CCL4, exceedingly expressed in the respiratory track and by blood PBMCs, further rising the cytokine storm (Xiong et al., 2020). Removing these pro-inflammatory signals from the blood reduces the uncontrolled activation of the immune cells and their recruitment to the targeted tissues. In addition to several treatments already in place or feasible with different efficiency and collateral effects (Ye et al., 2020), blood purification therapy has been successfully applied to some patients in critical condition of Covid19 (AL Shareef and Bakouri, 2020; Ma et al., 2020). Extracorporeal detoxification of the blood is an invasive method primarily used for sepsis to remove toxins and inflammatory cytokines (Monard et al., 2019). In recent years, nanotechnology has provided a variety of inorganic or organic materials with accidental or intended immunomodulatory properties (Feng et al., 2019). Among them, nanoparticles (NPs) with cytokine binding properties show great potential to quench the cytokine storm raised in Covid19 and other acute diseases, as well as NPs aiming at cytokine or chemokine intracellular signals and cognate receptors with antagonist capabilities.

## Discussion

The decrease of inflammatory mediators during a cytokine storm is therapeutically approached in three different ways: (a) downregulation of the cytokine expression; (b) antagonism of the cytokine cognate receptors; (c) physical removal of the cytokines (Ye et al., 2020). To impair cytokine release, common anti-inflammatory drugs (i.e., corticosteroids) or endogenous molecules (i.e., IL-λ) are used to modulate the pathways of cytokine-releasing cells. Targeting cytokine receptors is often performed using small molecules behaving as receptor antagonists; however, encouraging results have been obtained with monoclonal neutralizing antibodies. Particularly, the treatment of Covid19 patients with Tocilizumab, a recombinant humanized monoclonal antibody against the soluble and membrane-bound Interleukin-6 receptor (IL-6R), already approved for rheumatoid arthritis, successfully reduced the mortality rate (Zhang et al., 2020). Albeit clinically efficient, the removal of cytokines is often obtained by the blood purification techniques previously mentioned, which often require extracorporeal machinery and invasive protocols applied to the patients.

Among the theoretical treatment strategies to reduce Covid19 cytokine storm, the employment of cytokine binding NPs can be considered. Diverse materials show incidental sticking ability for cytokines and chemokines, like CXCL18 and TNF-α (Song et al., 2004; Brown et al., 2010; Tsai et al., 2012; Batt et al., 2018). In principle, the administration in the bloodstream of NPs with specific adsorption of inflammatory molecules would help their clearance. Unfortunately, many of those are metallic or carbon-based NPs, whose poor solubility and non-degradability induce their accumulation and persistency inside the body leading to cytotoxicity. NP size and surface charge should also be carefully considered, as their particular combination can induce fibrinogen unfolding, exacerbating the inflammatory condition (Deng et al., 2011). Furthermore, the competitive binding of several biomolecules showing similar NP-surface affinity and the protein devoted to the alien bodies' opsonization (e.g., complement proteins, pentraxins, natural antibodies) create a “protein corona” limiting the cytokine-binding particle efficiency (Ke et al., 2017). Yet, it is worth to mention that selected materials' adsorption is quite specific for some proteins, and the formation of a specific corona is also envisaged as a “natural” method for NP surface functionalization (Caracciolo, 2015; Palchetti et al., 2016; Caracciolo et al., 2017; Cagliani et al., 2019a).

Interesting results with potential clinical applications have been obtained by “*ad hoc*” NP surface modification aimed at binding/adsorbing specific cytokines (Isahak et al., 2016; Guryanov et al., 2017; Wang et al., 2019). An interesting example is represented by chemokine-binding polystyrene sulfonate brushes NPs that mimic the sulfated tyrosine residues of the N-terminal tail of the chemokine receptor (Isahak et al., 2016). Such NP functionalization results in binding affinity for CCL2 (previously known as MCP-1). CCL2 is the prototype of the inflammatory chemokine, strongly attracting monocyte/macrophages to sites of inflammation and highly expressed during Covid19 (Xiong et al., 2020). Even more selective nanotools to entrap specific chemokines are NPs behaving as synthetic decoy receptors (Guryanov et al., 2017). Heparin-coated polylactic acid (PLA) NPs bear on their surface two modified CCR5 fragments responsible for chemokine binding. The exploitation of chemokine interaction with these “nanotraps” released in the medium results in a convincing decrease of monocyte adhesion to human endothelial cells. It is worth to indicate that CCR5 is the cognate receptor for CCL5 (previously known as RANTES), CCL3 (MIP-1α) and CCL4 (MIP-1β). All these inflammatory chemokines are vastly produced in the respiratory track and in the blood during Covid19-induced cytokine storm, as reported above (Xiong et al., 2020). CCL2 and CCL5 adsorption has been also obtained with heparin coated micro and nanoparticles without further chemical moieties on the surface and applied for microdialysis *in vitro* and *in vivo* (Duo and Stenken, 2011; Giorgi-Coll et al., 2017). It is intriguing to think of a potential release of such NPs as injected intravenous formulation or respiratory spray to decrease the inflammatory mediators during the late stage of the disease. These NPs are synthesized using FDA-approved materials (i.e., PLA, PLGA) with proved biodegradability and negligible toxicity. In general, the respiratory tract offers many advantages for drug and NP delivery (Sung et al., 2007). It has a high surface area with rapid absorption due to wide vascularization, low thickness of the epithelial barrier and absence of the first-pass metabolism. Cytokines and chemokines overexpressed in the lungs during Covid19 could be quickly reached by the appropriate inhaled nanotools, entrapping them before their inflammatory signals were triggering an immune reaction. Furthermore, the same activated phagocytes contributing to the Covid19 damages would be effectively removing NPs with chemokines sequestered on the surface, lowering their concentration in the biological fluids. It is important to underline that detailed information for a successful delivery of inhaled NPs is still missing. The efficiency of targeting depends on particle size and density, which influence their deposition into the lung tissue (Paranjpe and Müller-Goymann, 2014). NPs with a diameter in the range of 100–300 nm can be reasonably deposited in the alveolar region, whereas it is still debated if NPs below a certain size could even be exhaled weakening the therapeutic efficacy (Yang et al., 2008). The opsonization mechanisms of the innate immune systems may likely change the NP size, so the surface chemistry should be properly designed and tested to target specific tissues. Anyway, the feasibility of intranasal treatment in ovalbumin-sensitized mice model for asthma has been proved with PEGylated dextran coated supermagnetic iron oxide nanoparticles (SPION) conjugated with anti-IL4Rα blocking antibodies (Halwani et al., 2016). IL4Rα is a shared subunit of IL-4 and IL3 receptors. The treatment with anti-IL4Rα NPs significantly decreased pro-inflammatory cytokine expression and release in broncho-alveolar lavage fluid (BALF) and lung tissue in mice. The activation of CD4 and CD8 T cells in the lung and their cytokine release were more efficiently impaired by anti-IL4Rα-NPs treatment than by free anti-IL4Rα antibodies.

Nanomaterials can also be employed to reduce the overstimulation of cytokines by inhibiting the signaling of their cognate receptors. We already mentioned that monoclonal antibodies like Tocilizumab are used as cytokine receptor antagonist to treat Covid19 (Zhang et al., 2020). Some biodegradable NPs are also under investigation to fulfill the same task and partially inhibit receptor signaling (Grozdanovic et al., 2019). Grozdanovich et al. described the effects of PEGylated R321-peptide NPs on eosinophil migration. This inhibitor peptide is derived from the second transmembrane helix of CCR3. R321 is able to self-assemble into uniform NPs and their administration inhibited CCR3-mediated chemotaxis of human blood eosinophils. Moreover, R321-NPs reduced eosinophil recruitment into the lung and airway hyper-responsiveness in a triple-allergen mouse asthma-model of allergic airway inflammation. The novelty of these NPs is related to their function as biased antagonists inhibiting only a part of the following signaling avoiding the β-arrestin-mediated receptor internalization and subsequent CCR3 degradation. The final outcome of removing inflammatory ligands by receptor internalization without triggering leukocyte migration would lower the inflammation.

Another way to antagonize the action of chemo-attractant cytokines could be obtained by the functionalization of NPs with the same chemokines covalently bound onto the particle surface (Cagliani et al., 2019b). We synthesized CXCL5-NPs proving that these particles are highly internalized in CXCR2^+^ cells but not in cells expressing low levels of this receptor. The described NP uptake removes CXCL5-cognate receptor from the cell surface making the leukocyte less responding to inflammatory stimuli. The research of chemokine-NP interactions with leukocytes is currently ongoing in our lab producing promising results with the use of biodegradable PLGA/pluronic NPs homogenously produced by microfluidic-assisted nanoprecipitation (Donno et al., 2017).

The firm adhesion of phagocytes to the endothelia in inflammatory diseases including Covid19 is mediated by chemokine-receptor-binding dependent conformational change of integrins increasing their affinity for specific ligands expressed on the endothelial cells (Mitroulis et al., 2015). Leukocyte transmigration can be also considered a therapeutic target and it has been investigated through the use of VCAM-1 glycoprotein-functionalized liposomes (Calin et al., 2015). VCAM-1 is upregulated on the endothelial cells and is the ligand for the integrins VLA-4 (CD49aCD29) and α_4_β_7_ (CD49dCD29), both expressed on monocytes/macrophages. Calin et al. demonstrated that VLA-4-liposomes with encapsulated a CCR2 antagonist were able to decrease chemokine-dependent inflammatory processes *in vitro* and *in situ* adding an integrin-ligand interaction as potential NP therapeutic target.

Like all the prolonged and uncontrolled inflammations, also Covid19 is characterized by redox unbalance and oxidative stress, especially in ARDS condition (Delgado-Roche and Mesta, 2020). The consistent production of reactive-oxygen species (ROS) (i.e., superoxide anion, hydrogen peroxide and hypochlorous acid) by cells involved in the immune response has a relevant role to worsen the effects of inflammation. Once released in the cytoplasm, ROS exacerbate inflammation by oxidizing protein and lipid cellular constituents, damaging DNA, and contributing to induce apoptosis. Neutrophils, M1 macrophages and endothelial cells produce ROS especially through NADH/NADPH oxidase (NOX) activation (Mittal et al., 2014). An interesting hypothesis by Baqi et al. (2020). highlights the possibility that the activation of Ca^2+^-sensitive Nox and ROS production can be further stimulated by Sars Cov 2 entry via angiotensin-converting enzyme 2 (ACE2) receptor, inducing the generation of angiotensin 1,7 from angiotensin II and the consequent opening of Ca^2+^ channels (Baqi et al., 2020). Covid19 could be also treated with anti-oxidants materials as complementary support to decrease inflammation. Nevertheless, the majority of these materials, such as flavonoids, carotenoids, and polyphenols regulating ROS production at different levels, often show low bioavailability, short time of circulation, poor target specificity and non-specific dispersion in normal tissues (Crascì et al., 2018). Efficient ROS scavenging activity has been obtained using inorganic nanomaterials with catalytic surface such as cerium oxide (CeO_2_) or platinum (Pt) NPs in endothelial cells (Del Turco et al., 2019) and monocytes (Gatto et al., 2018), respectively. For long term clinical applications, however, it could be preferable using very soluble and biodegradable NPs. A promising example is represented by novel PEGylated poly(propylene sulfide) (PPS) NPs (Rajkovic et al., 2019). PPS-NPs displayed significant ROS scavenging and anti-oxidant activity through a high density of sulfur atoms. Relevant reduction of TNFα and IL-6 expression, microglia and vascular activation have been demonstrated by the authors in a mouse model of stroke. Moreover, after intravenous administration, PPS-NPs accumulated specifically in ischemic brain regions and retained for at least 7 days, demonstrating their potential for an extended therapeutic action.

In our opinion, all the nanomaterials so far mentioned could find applications to reduce acute inflammation and cytokine storms occurring in Covid19 and related diseases. We also believe that this field of research will foster the availability of defense approaches for public health in the future.

## Author Contributions

AP, PP, and GB wrote and revised the paper. All authors contributed to the article and approved the submitted version.

## Conflict of Interest

The authors declare that the research was conducted in the absence of any commercial or financial relationships that could be construed as a potential conflict of interest.
